# HSPA4 Enhances BRSV Entry via Clathrin-Mediated Endocytosis Through Regulating the PI3K–Akt Signaling Pathway and ATPase Activity of HSC70

**DOI:** 10.3390/v16111784

**Published:** 2024-11-17

**Authors:** Yang Liu, Qiongyi Li, Shuai Shao, Xiaolan Ji, Wanning Gao, Yiyang Fan, Mingqi Liu, Yan Wang, Jialin Bai

**Affiliations:** 1 Key Laboratory of Biotechnology and Bioengineering of State Ethnic Affairs Commission, Biomedical Research Center, Northwest Minzu University, Lanzhou 730030, China; m15730981229@163.com (Y.L.); ss17695931107@163.com (S.S.); jxl18226033901@163.com (X.J.); gwn17679013680@163.com (W.G.); 13354925037@163.com (Y.F.); 13595842748@163.com (M.L.); wysakura616@163.com (Y.W.); 2 College of Life Science and Engineering, Northwest Minzu University, Lanzhou 730030, China

**Keywords:** bovine respiratory syncytial virus, heat shock protein family A member 4, heat shock cognate protein 70, clathrin, endocytosis

## Abstract

Bovine respiratory syncytial virus (BRSV) is an enveloped RNA virus that utilizes clathrin-mediated endocytosis for cell entry and is a significant pathogen in bovine respiratory disease (BRD). Heat shock protein family A member 4 (HSPA4), a member of the HSP70 family, is known to be involved in the progression of various cancers. However, its role in virus entry has not been previously explored. Through experiments involving Western blot analysis, virus titer, and virus copies analysis, we demonstrated that HSPA4 can regulate BRSV entry and replication. The specific regulation mode is to enhance BRSV entry by promoting clathrin-mediated endocytosis. We used Western blot, virus titer, virus copies analysis, and IFA to demonstrate that HSPA4 can promote clathrin heavy chain protein (CHC) expression and further promote BRSV entry by activating the PI3K–Akt signaling pathway. Furthermore, we observed that HSPA4 boosts the efficiency of clathrin-mediated endocytosis by increasing the ATPase activity of heat shock cognate protein 70 (HSC70), thereby facilitating BRSV entry. Additionally, our investigation into the impact of HSPA4 on the entry of other viruses revealed that HSPA4 can facilitate the entry of a variety of viruses into host cells.

## 1. Introduction

Bovine respiratory syncytial virus (BRSV), a member of the *Paramyxoviridae family* in the *genus Pneumovirus* [1], is an enveloped RNA virus that primarily affects the bovine respiratory tract, leading to bovine respiratory disease complex (BRDC) [1,2,3,4]. BRSV transmission occurs through nasal secretions, resulting in significant economic losses in the global cattle industry. The entry of the virus into host cells is a crucial step in the viral infection cycle, facilitating intracellular replication [5,6,7]. Endocytosis is the process through which cells internalize large molecules or small particles by encapsulation in the cell membrane. This mechanism is vital for nutrient uptake, processing foreign substances, and the regulation of cell surface molecule expression [8,9,10]. Viruses, including BRSV, utilize endocytosis by binding to specific receptors on the cell surface and triggering internalization [10,11]. The endocytic pathways, such as clathrin-mediated, caveolin-mediated, and macropinocytosis pathways, play a role in this process [10,11]. BRSV has been shown to enter MDBK cells through the clathrin-mediated endocytic pathway, which is regulated by the PI3K–Akt signaling pathway [12].

Heat shock protein family A member 4 (HSPA4) belongs to the heat shock protein 70 (HSP70) family and the heat shock protein 110 (HSP110) subfamily [13,14]. As a molecular chaperone, HSPA4 is involved in protein folding, assembly, transport, and the repair or degradation of damaged proteins, contributing to the maintenance of protein homeostasis [15,16,17,18]. HSPA4 is implicated in the pathogenesis of various diseases, particularly cancer, and its expression level is closely linked to tumor invasion and metastasis [19,20,21]. HSPA4 may impact tumor development through various mechanisms, including the modulation of cyclin and apoptosis-related proteins as well as the activation of the Akt signaling pathway [22]. However, the role of HSPA4 in virus infection is rarely reported. HSC70, another molecular chaperone protein, plays a key role in regulating clathrin-mediated endocytosis. Acting as a clathrin-uncoating ATPase, it interacts with clathrin to modulate the assembly and disassembly of clathrin coats, thereby enhancing the efficiency of the clathrin-mediated endocytosis pathway [23,24]. Furthermore, the binding of HSP110 to the ATPase of HSC70 increases the ATPase activity of HSC70, promoting the release of ADP and the combination with ATP and ultimately enhancing clathrin-mediated endocytosis [25,26].

On the basis of the above studies, we hypothesized that HSPA4 might be involved in BRSV entry into MDBK cells. Our research demonstrated that HSPA4 promotes BRSV entry into MDBK cells through clathrin-mediated endocytosis. Specifically, the activation of the PI3K–Akt signaling pathway by HSPA4 upregulates CHC expression, thereby increasing clathrin-mediated endocytosis and promoting BRSV entry. Additionally, HSPA4 strengthens HSC70 ATPase activity, leading to the release of clathrin and improving the efficiency of clathrin-mediated endocytosis, further enhancing BRSV entry.

## 2. Materials and Methods

### 2.1. Cells and Viruses

MDBK, Vero, and PK15 cells were obtained from the China Animal Health and Epidemiology Center and cultured in DMEM (Procell Life Science, Wuhan, China) supplemented with 10% FBS (Jinyuankang Bio-engineering, Guangzhou, China). The BRSV, bovine herpesvirus-1 (BoHV-1), bovine parainfluenza virus 3 (BPIV3), porcine epidemic diarrhea virus (PEDV), and transmissible gastroenteritis virus (TGEV) strains, were sourced from the China Animal Health and Epidemiology Center. The BRSV Yunnan strain, BoHV-1 Shaanxi strain, BPIV3 Yunnan strain, and PEDV Shandong strain were isolated and characterized in 2021, whereas the TGEV Shandong strain was isolated and characterized in 2019.

### 2.2. Antibodies and Reagents

Rabbit monoclonal antibody against PI3K p85 (phospho-Y458) + PI3 kinase p55 (phospho-Y199), mouse monoclonal antibody against GAPDH, rabbit monoclonal antibody against clathrin heavy chain (CHC), mouse monoclonal antibody against CHC, Alexa fluor-488-conjugated anti-rabbit, Cy™3-conjugated anti-mouse IgG (H+L), and mounting medium containing DAPI were purchased from Abcam (Abcam, Cambridge, UK). Rabbit monoclonal antibody against phospho-Akt (Ser473), rabbit monoclonal antibody against HSPA4, and mouse monoclonal antibody against HSC70 were purchased from Cell Signaling Technology (Cell Signaling Technology, Danvers, MA, USA). Wortmannin, apoptozole, VER 155008, and chlorpromazine were obtained from Sigma (Sigma, St. Louis, MO, USA). Akti-1/2 was obtained from Abcam (Abcam, Cambridge, UK).

### 2.3. Plasmid and siRNA Transfections

The plasmids utilized in this research were supplied by the China Animal Health and Epidemiology Center, while the siRNAs were specifically crafted and produced by Tsingke Biotechnology (Tsingke Biotechnology, Beijing, China). MDBK cells were initially plated in 6-well cell culture plates. Upon reaching a cell confluence of 70–80%, either plasmids or siRNAs were transfected into the MDBK cells following the Lipofectamine™ 3000 (ThermoFisher, Waltham, MA, USA) guidelines. The impact of protein overexpression or silencing was assessed via Western blot analysis 24 h post-transfection. The siRNAs used in this experiment are shown in Table 1.

### 2.4. Western Blot and Gray Scale Analysis

Cells were lysed in radioimmunoprecipitation assay (RIPA) buffer containing 1% phenylmethylsulfonyl fluoride (PMSF) and heated at 95 °C for 10 min. The lysate was then run on a 10% SDS-PAGE gel and transferred to polyvinylidene fluoride (PVDF) membranes. Following treatment with 5% skim milk powder for 1 h, the cells were incubated with primary antibodies overnight at 4 °C. The samples were subsequently incubated with horseradish peroxidase (HRP)-labeled goat anti-rabbit/mouse secondary antibody for 1 h at room temperature. Bands on the membrane were analyzed using a fluorescence chemiluminescence analyzer (UVITEC, Cambridge, England). The target bands were subjected to gray scale analysis using Image J 1.54d.

### 2.5. Virus Replication

MDBK cells were incubated with BRSV at a multiplicity of infection (MOI) of 2 for 1 h at 4 °C to allow for virus attachment to the cell surface. The cells were then washed multiple times with PBS to remove any unabsorbed BRSV particles. After 24 h of culture at 37 °C in DMEM containing 2% FBS, RNA was extracted and the number of viral copies was determined by qPCR. Additionally, culture supernatant collected at 24 h was used for TCID_50_ detection.

### 2.6. Virus Attachment

MDBK cells were incubated with BRSV at a multiplicity of infection (MOI) of 10 for 1 h at 4 °C to allow for virus attachment to the cell surface. The cells were then washed multiple times with PBS to remove any unabsorbed BRSV particles. The attached viruses were collected by repeated freezing and thawing at −80 °C for the virus titer and virus copy analyses.

### 2.7. Virus Entry

MDBK cells were incubated with BRSV at a multiplicity of infection (MOI) of 2 for 1 h at 4 °C to allow for virus attachment to the cell surface. The cells were then washed multiple times with PBS to remove any unabsorbed BRSV particles. After the cells were incubated for 1 h at 37 °C, proteinase K was applied at 4 °C to eliminate any remaining extracellular virus particles, followed by treatment with 2 mM phenylmethylsulfonyl fluoride (PMSF). Subsequently, RNA extraction and reverse transcription were performed to analyze virus entry using qPCR.

### 2.8. TCID_50_ Analysis

MDBK cells were seeded in 96-well plates 24 h prior to the experiment. Following reaching full confluence, the virus was serially diluted by a factor of 10 and then seeded into 96-well plates. The cells were incubated at 37 °C for 1 h, washed multiple times with PBS, and then supplemented with 2% FBS DMEM. The TCID_50_ was determined using the Reed–Muench formula [27].

### 2.9. qPCR

Infected cells were collected and RNA was extracted, followed by reverse transcription. A real-time PCR system (TIANLONG, Xian, China) was utilized to detect BRSV copies. The primers and probes used for BRSV-N detection can be found in Table 1. The number of BRSV copies was calculated as follows: 10^(47.686-Ct)/3.466^.

### 2.10. Cell Viability Determination

MDBK cells were seeded into 96-well plates and treated with an inhibitor for 24 h after reaching full confluence. Subsequently, CCK-8 reagent (Vazyme Biotech, Nanjing, China) was added. Following a 4 h incubation at 37 °C, the absorbance was assessed at 450 nm using a microplate reader (Flash Spectrum Biotechnology, Shanghai, China).

### 2.11. Immunofluorescence

The infected cells were treated with 75% ethanol for 1 h at 4 °C. Following a PBS wash, the cells were exposed to a primary antibody at 37 °C for 1 h. Afterward, the cells were washed again and incubated with a secondary antibody for 1 h at 37 °C. Finally, the cells were fixed using mounting medium containing DAPI and examined under a Leica STELLARIS 5 confocal microscope (Leica, Frankfurt, Germany).

### 2.12. Co-Immunoprecipitation (Co-IP)

Cells were collected and lysed in radioimmunoprecipitation assay (RIPA) buffer. Subsequently, 1 μg of a primary antibody was added to 400 μL of cell lysate and incubated overnight at 4 °C. Next, 20 μL of protein A+G agarose was added, and the mixture was gently shaken for 3 h at 4 °C before centrifugation. The supernatant was then carefully removed. The precipitate was washed five times with PBS, after which, SDS-PAGE electrophoresis loading buffer was added and the sample was heated at 95 °C for 10 min to prepare for Western blot analysis.

### 2.13. Statistical Analyses

The experimental results were obtained from three separate groups of experiments and analyzed using one-way ANOVA in GraphPad Prism Version 5.0. Significant differences in the data are denoted as * *p* < 0.05, ** *p* < 0.01, and *** *p* < 0.001.

## 3. Results

### 3.1. HSPA4 Promotes BRSV Replication in MDBK Cells

**Figure 1:**
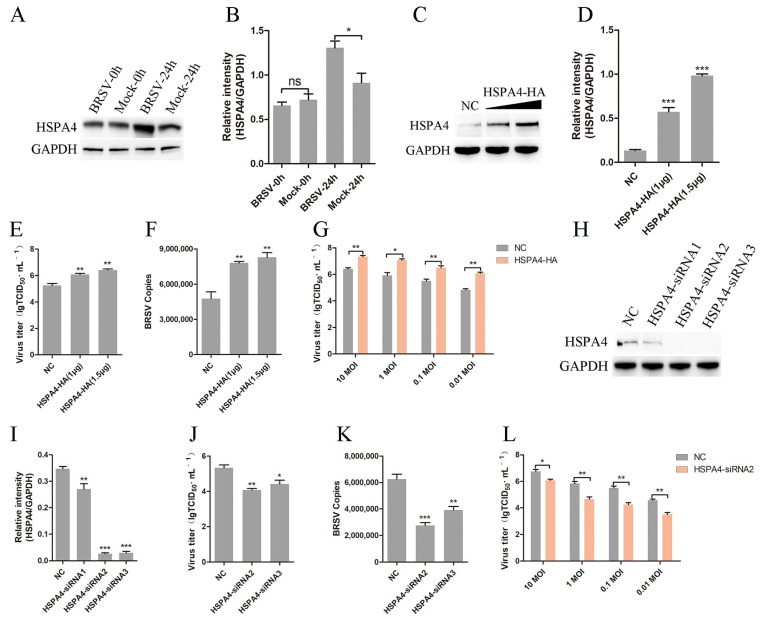
HSPA4 is involved in BRSV replication. (**A**,**B**) MDBK cells infected with BRSV for 0 h or 24 h were collected, and HSPA4 expression was detected by Western blot and gray scale analysis. (**C**,**D**) MDBK cells were transfected with 1.0 μg or 1.5 μg of HSPA4-HA plasmid, and the cells were collected 24 h later to detect HSPA4 expression by Western blot and gray scale analysis. (**E**) MDBK cells were transfected with 1.0 μg or 1.5 μg of HSPA4-HA plasmid, bound with BRSV at an MOI of 2, transferred to 4 °C for 1 h, and then transferred to 37 °C for 24 h. The supernatant was collected, and BRSV replication was analyzed by quantifying the virus titer; (**F**) the cells were collected, and BRSV replication was analyzed by quantifying the number of virus copies. (**G**) MDBK cells were transfected with 1.5 μg of HSPA4-HA plasmids, bound with different doses of BRSV, transferred to 4 °C for 1 h, and then transferred to 37 °C for 24 h. The supernatant was collected and BRSV replication was analyzed by quantifying the virus titer. (**H**,**I**) MDBK cells were transfected with HSPA4-siRNA1, HSPA4-siRNA2 or HSPA4-siRNA3, and the cells were collected 24 h later to detect HSPA4 expression by Western blot and gray scale analysis. (**J**) MDBK cells were transfected with HSPA4-siRNA2 or HSPA4-siRNA3, bound with BRSV at an MOI of 2, transferred to 4 °C for 1 h, and then transferred to 37 °C for 24 h. The supernatant was collected, and BRSV replication was analyzed by quantifying the virus titer; (**K**) the cells were collected, and BRSV replication was analyzed by quantifying the number of virus copies. (**L**) MDBK cells were transfected with HSPA4-siRNA2, bound with different doses of BRSV, transferred to 4 °C for 1 h, and then transferred to 37 °C for 24 h. The supernatant was collected, and BRSV replication was analyzed by quantifying the virus titer. *, *p* < 0.05; **, *p* < 0.01; ***, *p* < 0.001; ns, *p* > 0.05.

To investigate the role of HSPA4 in BRSV replication, MDBK cells were infected with BRSV for 0 h or 24 h, and HSPA4 expression was detected. The results of the Western blot and gray scale analysis showed that BRSV infection promoted HSPA4 expression (Figure 1A,B). We transfected MDBK cells with an HSPA4-HA plasmid and subsequently inoculated them with BRSV for 24 h to assess the impact of HSPA4 overexpression on BRSV replication. The Western blot analysis and gray scale analysis revealed a dose-dependent increase in HSPA4 expression in MDBK cells transfected with the HSPA4-HA plasmid (Figure 1C,D). MDBK cells were transfected with the HSPA4-HA plasmid and infected with BRSV, and the progeny virus titer and virus copies also presented dose-dependent increases (Figure 1E,F). Additionally, we examined the effects of varying the BRSV infection dose on viral replication in the presence of HSPA4 overexpression. The virus titer results demonstrated that HSPA4 overexpression facilitated BRSV replication at different infection doses (Figure 1G). The transfection of three different HSPA4 siRNAs into MDBK cells revealed that siRNA2 and siRNA3 effectively silenced HSPA4 (Figure 1H,I). Next, we inoculated the cells with BRSV for 24 h after the HSPA4 siRNA transfection; the viral titer and viral copy results revealed that silencing HSPA4 inhibited BRSV replication in MDBK cells (Figure 1J,K). We inoculated cells with BRSV at different doses after silencing HSPA4, and the results showed that HSPA4 silencing inhibited BRSV replication at different virus inoculation doses (Figure 1L). Collectively, these findings suggest that HSPA4 promotes BRSV replication in MDBK cells.

### 3.2. HSPA4 Promotes BRSV Entry into MDBK Cells

**Figure 2:**
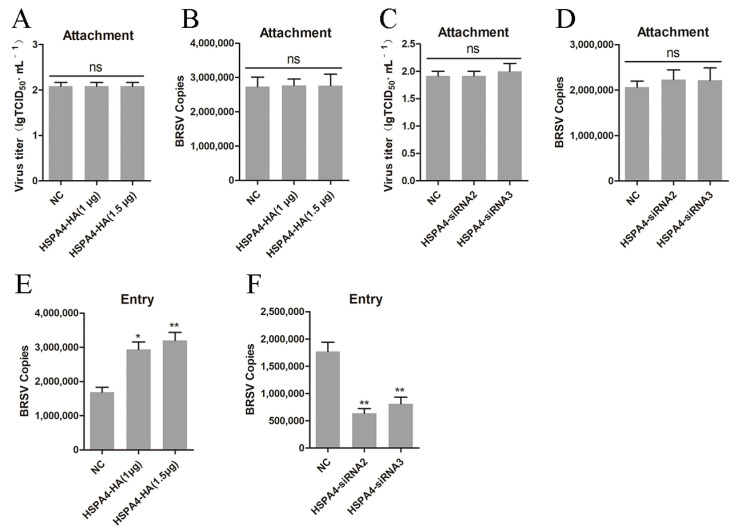
HSPA4 is involved in BRSV entry instead of attachment. (**A**) MDBK cells were transfected with 1.0 μg or 1.5 μg of HSPA4-HA plasmid and bound with BRSV at an MOI of 10 at 4 °C for 1 h; the attached virus was collected by repeated freezing and thawing at −80 °C for virus titer analysis (**B**) and virus copy analysis. (**C**) MDBK cells were transfected with HSPA4-siRNA2 or HSPA4-siRNA3 and bound with BRSV at an MOI of 10 at 4 °C for 1 h; the attached virus was collected by repeated freezing and thawing at −80 °C for virus titer analysis (**D**) and virus copy analysis. (**E**) MDBK cells were transfected with 1.0 μg or 1.5 μg of HSPA4-HA plasmid, bound with BRSV at an MOI of 2, transferred to 4 °C for 1 h, and then transferred to 37 °C for 1 h. The cells were collected, and BRSV entry was analyzed by quantifying the number of virus copies. (**F**) MDBK cells were transfected with HSPA4-siRNA2 or HSPA4-siRNA3, bound with BRSV at an MOI of 2, transferred to 4 °C for 1 h, and then transferred to 37 °C for 1 h. The cells were collected, and BRSV entry was analyzed by quantifying the number of virus copies. *, *p* < 0.05; **, *p* < 0.01; ns, *p* > 0.05.

We investigated the impact of HSPA4 on BRSV attachment. MDBK cells were transfected with the HSPA4-HA plasmid and then inoculated with BRSV at 4 °C for 1 h to assess virus attachment. The virus titer and virus copy results indicated that overexpression of HSPA4 did not influence BRSV attachment (Figure 2A,B). Similarly, when HSPA4 was silenced in MDBK cells, the virus titer and virus copy results suggested that BRSV attachment remained unaffected (Figure 2C,D). Furthermore, we explored the role of HSPA4 in BRSV entry into cells. After transfecting the HSPA4-HA plasmid into MDBK cells and inoculating them with BRSV at 4 °C for 1 h followed by a shift to 37 °C for 1 h to detect virus entry, the analysis of the number of viral copies revealed that HSPA4 overexpression facilitated BRSV entry (Figure 2E). Conversely, silencing HSPA4 hindered BRSV entry (Figure 2F). These findings suggest that HSPA4 can enhance the entry of BRSV into MDBK cells.

### 3.3. HSPA4 Promotes BRSV Entry into MDBK Cells Through Clathrin-Mediated Endocytosis

**Figure 3:**
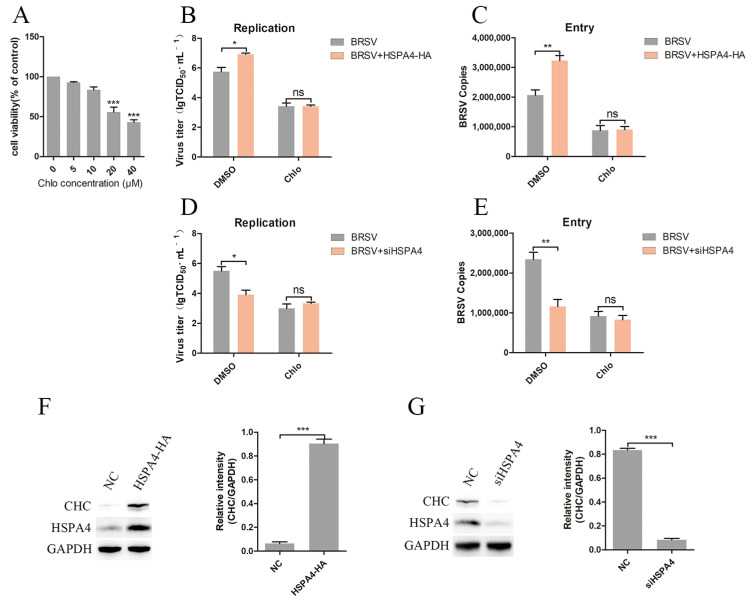
HSPA4 promotes clathrin-mediated endocytosis. (**A**) MDBK cells were treated with different concentrations of chlorpromazine for 24 h and analyzed using CCK-8 reagent to detect cell viability. (**B**,**D**) MDBK cells were transfected with HSPA4-HA plasmid or HSPA4-siRNA2 (siHSPA4) and treated with DMSO or chlorpromazine for 1 h before infecting them with BRSV for 24 h; the supernatant was collected and BRSV replication was analyzed by quantifying the virus titer. (**C**,**E**) MDBK cells were transfected with HSPA4-HA plasmid or HSPA4-siRNA2 (siHSPA4) and treated with DMSO or chlorpromazine for 1 h before infecting them with BRSV for 1 h; the cells were collected and BRSV entry was analyzed by quantifying the number of virus copies. (**F**,**G**) MDBK cells were transfected with HSPA4-HA plasmids or HSPA4-siRNA2 (siHSPA4), and the cells were collected 24 h later to detect CHC and HSPA4 expression using Western blot and gray scale analysis. *, *p* < 0.05; **, *p* < 0.01; ***, *p* < 0.001; ns, *p* > 0.05.

BRSV can infect MDBK cells through clathrin-mediated endocytosis [12]. We next investigated the role of HSPA4 in regulating this pathway. We initially determined the appropriate concentration of chlorpromazine, an inhibitor of clathrin-mediated endocytosis, by assessing its impact on cell viability. The cell viability assays showed that chlorpromazine at a concentration greater than 10 μM inhibited cell viability (Figure 3A). We then transfected MDBK cells with the HSPA4-HA plasmid and treated them with DMSO or chlorpromazine before infecting them with BRSV. Our virus titer results revealed that overexpression of HSPA4 increased BRSV replication, which was counteracted by the chlorpromazine treatment (Figure 3B). The viral copy results showed that chlorpromazine inhibited the enhancement of BRSV entry caused by HSPA4 overexpression (Figure 3C). Conversely, silencing HSPA4 inhibited BRSV replication and entry, and this inhibitory effect was reversed by chlorpromazine (Figure 3D,E). Additionally, we examined the influence of HSPA4 on the expression of clathrin heavy chain protein (CHC), which is an important functional domain of clathrin [28,29]. Our Western blot and gray scale analysis findings demonstrated that overexpression of HSPA4 promoted CHC expression (Figure 3F), and silencing HSPA4 inhibited CHC expression (Figure 3G). Overall, our results indicate that HSPA4 promotes BRSV entry into MDBK cells via clathrin-mediated endocytosis.

### 3.4. HSPA4 Promotes CHC Expression and BRSV Entry by Regulating the PI3K–Akt Signaling Pathway

**Figure 4:**
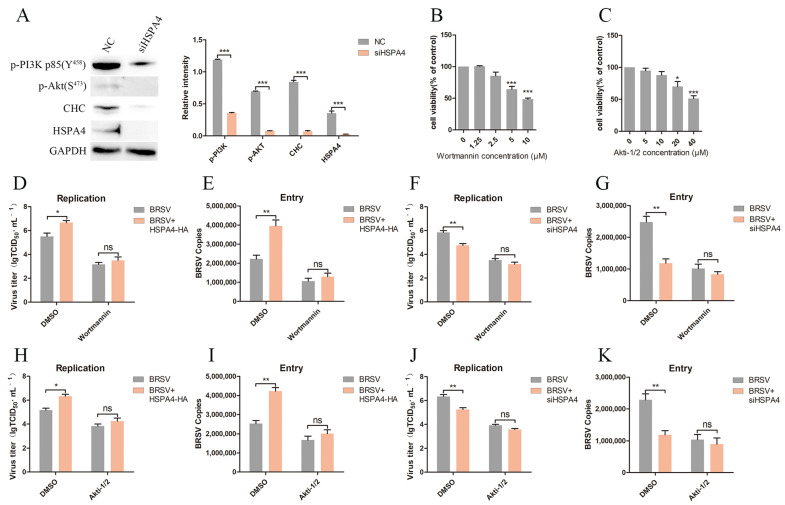
HSPA4 regulates PI3K–Akt signaling pathway. (**A**) MDBK cells were transfected with HSPA4-siRNA2 (siHSPA4) and the cells were collected 24 h later to detect p-PI3K, p-Akt, CHC, and HSPA4 expression by Western blot and gray scale analysis. (**B**,**C**) MDBK cells were treated with different concentrations of wortmannin or Akti-1/2 for 24 h, and analyzed using CCK-8 reagent to detect cell viability. (**D**,**H**) MDBK cells were transfected with HSPA4-HA plasmid and treated with DMSO, wortmannin, or Akti-1/2 for 1 h before infecting them with BRSV for 24 h; the supernatant was collected and BRSV replication was analyzed by quantifying the virus titer. (**E**,**I**) MDBK cells were transfected with HSPA4-HA plasmid and treated with DMSO, wortmannin, or Akti-1/2 for 1 h before infecting them with BRSV for 1 h; the cells were collected and BRSV entry was analyzed by quantifying the number of virus copies. (**F**,**J**) MDBK cells were transfected with HSPA4-siRNA2 (siHSPA4) and treated with DMSO, wortmannin, or Akti-1/2 for 1 h before infecting them with BRSV for 24 h; the supernatant was collected and BRSV replication was analyzed by quantifying the virus titer. (**G**,**K**) MDBK cells were transfected with HSPA4-siRNA2 (siHSPA4) and treated with DMSO, wortmannin, or Akti-1/2 for 1 h before infecting them with BRSV for 1 h; the cells were collected and BRSV entry was analyzed by quantifying the number of virus copies. *, *p* < 0.05; **, *p* < 0.01; ***, *p* < 0.001; ns, *p* > 0.05.

Previous studies have indicated that the PI3K–Akt signaling pathway is activated during BRSV infection, leading to increased CHC expression and BRSV entry [12]. Additionally, HSPA4 can regulate the Akt signaling pathway in U251 cells [22]. In this study, we investigated the impact of HSPA4 on the PI3K–Akt signaling pathway in MDBK cells. We silenced HSPA4 in MDBK cells and assessed the expression of p-PI3K, p-Akt, and CHC. The Western blot and gray scale analysis revealed that silencing HSPA4 reduced the levels of p-PI3K, p-Akt, and CHC (Figure 4A). We further explored the mechanism through which HSPA4 exerts its effect using wortmannin and Akti-1/2, inhibitors of PI3K and Akt, respectively. The cell viability assay results demonstrated that wortmannin at concentrations greater than 2.5 μM and Akti-1/2 at concentrations greater than 10 μM decreased MDBK cell viability (Figure 4B,C). We subsequently transfected MDBK cells with the HSPA4-HA plasmid, pretreated them with DMSO or wortmannin, and then inoculated them with BRSV to evaluate virus replication and entry. The results indicated that wortmannin counteracted the enhancing effect of HSPA4 overexpression on BRSV replication and entry (Figure 4D,E). Following HSPA4 silencing in MDBK cells, the cells were treated with DMSO or wortmannin before BRSV inoculation. The results revealed that wortmannin reversed the inhibitory effect of silencing HSPA4 on BRSV replication and entry (Figure 4F,G). Additionally, we investigated the role of Akt in the HSPA4-mediated regulation of BRSV replication and entry. Notably, Akti-1/2 abolished the promoting effect of HSPA4 overexpression on BRSV replication and entry (Figure 4H,I), and reversed the inhibition effect of silencing HSPA4 on BRSV replication and entry (Figure 4J,K). These findings highlight that HSPA4 promotes CHC expression and BRSV entry by regulating the PI3K–Akt signaling pathway.

### 3.5. HSPA4 Enhances BRSV Entry into MDBK Cells via Clathrin-Mediated Endocytosis Through Regulating the ATPase Activity of HSC70

**Figure 5:**
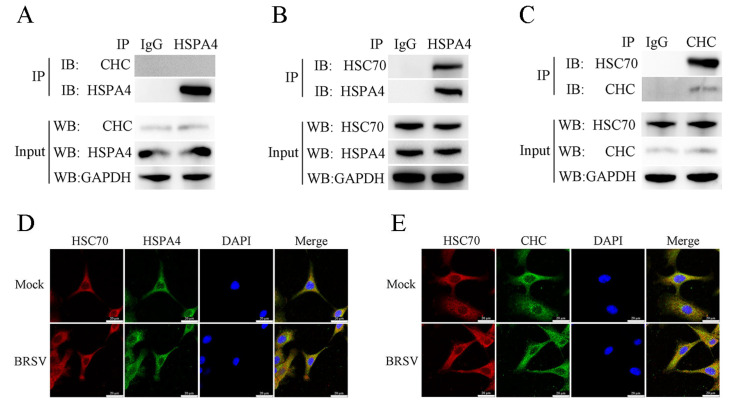
HSPA4 can interact with HSC70 and CHC can interact with HSP70. (**A**) MDBK cells were lysed and immunoprecipitated with anti-HSPA4 or IgG antibodies. The total cell lysates were analyzed with anti-CHC, anti-HSPA4, and anti-GAPDH antibodies. (**B**) MDBK cells were lysed and immunoprecipitated with anti-HSPA4 or IgG antibodies. The total cell lysates were analyzed with anti-HSC70, anti-HSPA4, and anti-GAPDH antibodies. (**C**) MDBK cells were lysed and immunoprecipitated with anti-CHC or IgG antibodies. The total cell lysates were analyzed with anti-HSC70, anti-CHC, and anti-GAPDH antibodies. (**D**) MDBK cells were infected with BRSV at an MOI of 5 and incubated for 1 h at 4 °C, and then incubated for 30 min at 37 °C. Confocal microscope analysis of HSC70 (red), HSPA4 (green), and cell nuclei (blue) in MDBK cells. Scale bar = 20 µm. (**E**) MDBK cells were infected with BRSV at an MOI of 5 and incubated for 1 h at 4 °C, and then incubated for 30 min at 37 °C. Confocal microscope analysis of HSC70 (red), CHC (green), and cell nuclei (blue) in MDBK cells. Scale bar = 20 µm.

**Figure 6:**
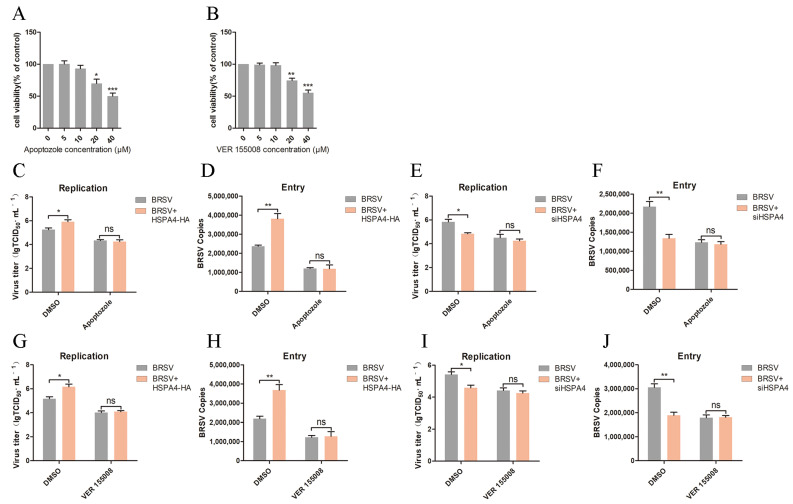
HSPA4 regulates the ATPase activity of HSC70. (**A**,**B**) MDBK cells were treated with different concentrations of apoptozole or VER155008 for 24 h and analyzed using CCK-8 reagent to detect cell viability. (**C**,**G**) MDBK cells were transfected with HSPA4-HA plasmid and treated with DMSO, apoptozole, or VER155008 for 1 h before infecting them with BRSV for 24 h; the supernatant was collected and BRSV replication was analyzed by quantifying the virus titer. (**D**,**H**) MDBK cells were transfected with HSPA4-HA plasmid and treated with DMSO, apoptozole, or VER155008 for 1 h before infecting them with BRSV for 1 h; the cells were collected and BRSV entry was analyzed by quantifying the number of virus copies. (**E**,**I**) MDBK cells were transfected with HSPA4-siRNA2 (siHSPA4) and treated with DMSO, apoptozole, or VER155008 for 1 h before infecting them with BRSV for 24 h; the supernatant was collected and BRSV replication was analyzed by quantifying the virus titer. (**F**,**J**) MDBK cells were transfected with HSPA4-siRNA2 (siHSPA4) and treated with DMSO, apoptozole, or VER155008 for 1 h before infecting them with BRSV for 1 h; the cells were collected and BRSV entry was analyzed by quantifying the number of virus copies. *, *p* < 0.05; **, *p* < 0.01; ***, *p* < 0.001; ns, *p* > 0.05.

HSC70 (heat shock cognate 70) can interact with CHC in HEK293T cells [30]. It releases ADP and binds ATP, further promoting the release of clathrin and improving the efficiency of clathrin-mediated endocytosis [30]. HSP110 is involved in this process; it can promote ADP release from and ATP binding to HSC70 [23,26]. Our study aimed to investigate whether HSPA4, a protein in HSP110 subfamily, could also play a role in promoting clathrin-mediated endocytosis and enhancing BRSV entry through this process in MDBK cells. Through co-immunoprecipitation assays, we confirmed that endogenous HSPA4 and CHC do not interact (Figure 5A), indicating that HSPA4 regulates CHC expression indirectly via the PI3K–Akt signaling pathway. However, we observed that endogenous HSPA4 and HSC70 can interact in MDBK cells, as can CHC and HSC70 (Figure 5B,C). Furthermore, our analysis of intracellular localization using immunofluorescence assays (IFAs) and confocal microscopy revealed significant co-localization of endogenous HSPA4 and HSC70 regardless of BRSV infection (Figure 5D); co-localization of HSC70 and CHC was also evident (Figure 5E). We used apoptozole and VER155008, which are inhibitors of HSC70 ATPase activity [31,32], to further validate our hypothesis. The cell viability assay results demonstrated that concentrations higher than 10 μM for apoptozole and VER155008 decreased MDBK cell viability (Figure 6A,B). We subsequently transfected MDBK cells with the HSPA4-HA plasmid and treated them with DMSO or apoptozole before BRSV inoculation to assess virus replication and entry. Indeed, apoptozole eliminated the enhancement of BRSV replication and entry caused by HSPA4 overexpression (Figure 6C,D). When HSPA4 was silenced, apoptozole eliminated the reduction in BRSV replication and entry caused by HSPA4 silencing (Figure 6E,F). These findings suggest that HSPA4 facilitates BRSV entry through clathrin-mediated endocytosis by modulating the ATPase activity of HSC70. The results using VER155008 further supported this conclusion (Figure 6G–J).

### 3.6. HSPA4 Promotes the Entry of a Variety of Viruses by Enhancing Clathrin-Mediated Endocytosis

**Figure 7:**
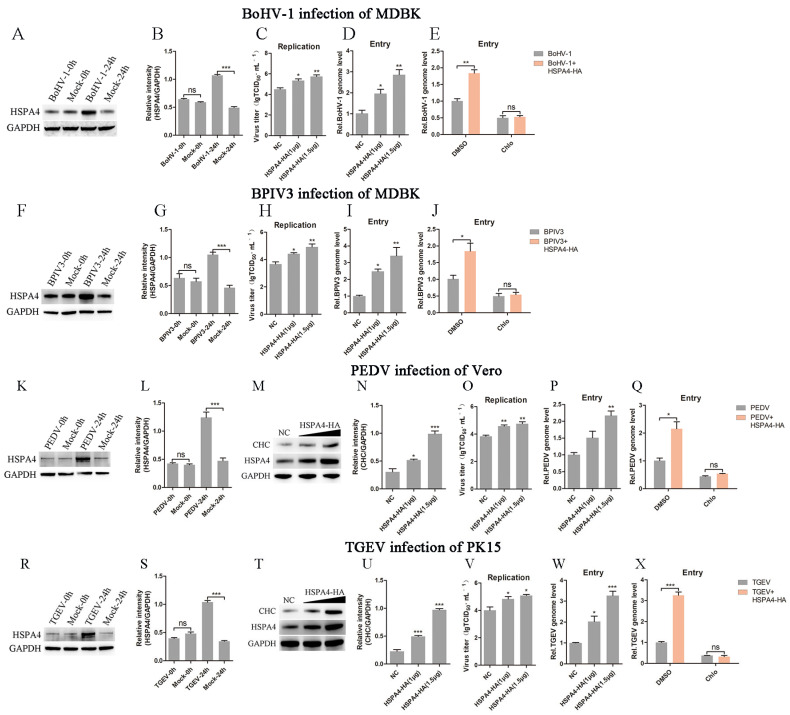
HSPA4 promotes the entry of a variety of viruses. (**A**,**B**) MDBK cells were infected with BoHV-1 at an MOI of 2 for 0 h or 24 h, and HSPA4 expression was detected by Western blot and gray scale analysis. (**C**) MDBK cells were transfected with 1.0 μg or 1.5 μg of HSPA4-HA plasmid, bound with BoHV-1 at an MOI of 2, transferred to 4 °C for 1 h, and then transferred to 37 °C for 24 h. The supernatant was collected and BoHV-1 replication was analyzed by quantifying the virus titer; (**D**) the cells were transferred to 37 °C for 1 h, and BoHV-1 entry was analyzed by quantifying the number of virus copies. (**E**) MDBK cells were transfected with HSPA4-HA plasmid and treated with DMSO or chlorpromazine for 1 h before infecting them with BoHV-1 for 1 h; the cells were collected and BoHV-1 entry was analyzed by quantifying the number of virus copies. (**F**,**G**) MDBK cells were infected with BPIV3 at an MOI of 2 for 0 h or 24 h, and HSPA4 expression was detected by Western blot and gray scale analysis. (**H**) MDBK cells were transfected with 1.0 μg or 1.5 μg of HSPA4-HA plasmid, bound with BPIV3 at an MOI of 2, transferred to 4 °C for 1 h, and then transferred to 37 °C for 24 h. The supernatant was collected and BPIV3 replication was analyzed by quantifying the virus titer; (**I**) the cells were transferred to 37 °C for 1 h and BPIV3 entry was analyzed by quantifying the number of virus copies. (**J**) MDBK cells were transfected with HSPA4-HA plasmid and treated with DMSO or chlorpromazine for 1 h before infecting them with BPIV3 for 1 h; the cells were collected and BPIV3 entry was analyzed by quantifying the number of virus copies. (**K**,**L**) Vero cells were infected with PEDV at an MOI of 2 for 0 h or 24 h, and HSPA4 expression was detected by Western blot and gray scale analysis. (**M**,**N**) Vero cells were transfected with 1.0 μg or 1.5 μg of HSPA4-HA plasmid, and the cells were collected 24 h later to detect CHC and HSPA4 expression by Western blot and gray scale analysis. (**O**) Vero cells were transfected with 1.0 μg or 1.5 μg of HSPA4-HA plasmid, bound with PEDV at an MOI of 2, transferred to 4 °C for 1 h, and then transferred to 37 °C for 24 h. The supernatant was collected and PEDV replication was analyzed by quantifying the virus titer; (**P**) the cells were transferred to 37 °C for 1 h and PEDV entry was analyzed by quantifying the number of virus copies. (**Q**) Vero cells were transfected with HSPA4-HA plasmid and treated with DMSO or chlorpromazine for 1 h before infecting them with PEDV for 1 h; the cells were collected and PEDV entry was analyzed by quantifying the number of virus copies. (**R**,**S**) PK15 cells were infected with TGEV at an MOI of 2 for 0 h or 24 h, and HSPA4 expression was detected by Western blot and gray scale analysis. (**T**,**U**) PK15 cells were transfected with 1.0 μg or 1.5 μg of HSPA4-HA plasmid, and the cells were collected 24 h later to detect CHC and HSPA4 expression by Western blot and gray scale analysis. (**V**) PK15 cells were transfected with 1.0 μg or 1.5 μg of HSPA4-HA plasmid, bound with TGEV at an MOI of 2, transferred to 4 °C for 1 h, and then transferred to 37 °C for 24 h. The supernatant was collected and TGEV replication was analyzed by quantifying the virus titer; (**W**) the cells were transferred to 37 °C for 1 h and TGEV entry was analyzed by quantifying the number of virus copies. (**X**) PK15 cells were transfected with HSPA4-HA plasmid and treated with DMSO or chlorpromazine for 1 h before infecting them with TGEV for 1 h; the cells were collected and TGEV entry was analyzed by quantifying the number of virus copies. *, *p* < 0.05; **, *p* < 0.01; ***, *p* < 0.001; ns, *p* > 0.05.

In order to test whether HSPA4 can promote the entry of other viruses that rely on clathrin-mediated endocytosis, we detected the effects of HSPA4 overexpression on the entry of bovine herpesvirus-1 (BoHV-1), bovine parainfluenza virus 3 (BPIV3), porcine epidemic diarrhea virus (PEDV), and transmissible gastroenteritis virus (TGEV) into cells [30,33,34,35]. To investigate the role of HSPA4 in BRSV replication, MDBK cells were infected with BoHV-1 or BPIV3 for 0 h or 24 h, and HSPA4 expression was detected. The results of the Western blot and gray scale analysis showed that BoHV-1 or BPIV3 infection promoted HSPA4 expression (Figure 7A,B,F,G). After overexpressing HSPA4 in MDBK cells, we infected the cells with BoHV-1 or BPIV3 for 24 h to detect virus replication and infected the cells with BoHV-1 or BPIV3 for 1 h to detect virus entry. The virus titers revealed that overexpressing HSPA4 inhibited BoHV-1 and BPIV3 replication (Figure 7C,H), while the number of virus copies revealed that overexpressing HSPA4 inhibited the entry of BoHV-1 or BPIV3 (Figure 7D,I). We transfected MDBK cells with the HSPA4-HA plasmid and treated them with DMSO or chlorpromazine before infecting them with BoHV-1 or BPIV3. Our results of virus copies revealed that overexpression of HSPA4 increased BoHV-1 or BPIV3 entry, which was counteracted by chlorpromazine treatment (Figure 7E,J). MDBK cells were infected with PEDV for 0 h or 24 h, and HSPA4 expression was detected. The results of the Western blot and gray scale analysis showed that PEDV infection promoted HSPA4 expression (Figure 1K,L). We detected CHC expression after overexpressing HSPA4 in Vero cells and the results revealed that HSPA4 overexpression could promoted CHC expression in Vero cells (Figure 7M,N). Vero cells were inoculated with PEDV after overexpressing HSPA4 to detect virus replication and entry. The virus titer and virus copy results showed that HSPA4 overexpression could increase PEDV replication and entry into Vero cells (Figure 7O,P). We transfected MDBK cells with the HSPA4-HA plasmid and treated them with DMSO or chlorpromazine before infecting them with PEDV. The number of virus copies revealed that chlorpromazine eliminated the enhancement of PEDV entry caused by HSPA4 overexpression (Figure 7Q). MDBK cells were infected with PEDV and HSPA4 expression was detected; the results showed that TGEV infection promoted HSPA4 expression (Figure 7R,S). We also found that CHC expression was increased after overexpressing HSPA4 in PK15 cells (Figure 7T,U). PK15 cells were inoculated with TGEV after HSPA4 was overexpressed to detect virus replication and entry. The results revealed that HSPA4 overexpression could promote TGEV replication and entry (Figure 7V,W). Chlorpromazine also eliminated the enhancement of TGEV entry caused by HSPA4 overexpression (Figure 7X). These results indicate that HSPA4 can promote the entry of a variety of viruses by enhancing clathrin-mediated endocytosis.

## 4. Discussion

BRSV is a significant pathogen that belongs to the *Paramyxoviridae* family, specifically the *Pneumovirus* genus [1]. It is an enveloped virus known to infect both the upper and lower respiratory tract, and is primarily excreted through nasal secretions. BRSV predominantly affects the respiratory system of cattle and is a key contributor to bovine respiratory disease complex (BRDC) [1,2,3,4]. Upon infection, the virus enters the host cell cytoplasm to replicate, marking a crucial stage in its life cycle [5,6,7]. Through endocytosis, a cellular mechanism for absorbing external materials, various viruses can gain entry into host cells [10,11]. Endocytosis pathways, such as clathrin-mediated endocytosis, caveolae-mediated endocytosis, and macropinocytosis, play a role in viral entry [10,11]. Previous studies have shown that BRSV enters MDBK cells through clathrin-mediated endocytosis [12]. HSPA4, a member of the HSP70 family and part of the HSP110 subfamily, is found in the cytoplasm, lipid droplets, and nuclei [36]. It is associated with the PI3K–Akt signaling pathway, which plays a crucial role in tumor cell proliferation, survival, and aggressiveness [22]. Despite this, there have been no previous studies on the involvement of HSPA4 in virus entry. This study investigated the role of HSPA4 in regulating BRSV entry. The findings suggest that HSPA4 promotes clathrin expression by activating the PI3K–Akt pathway, thereby facilitating BRSV entry through clathrin-mediated endocytosis. Furthermore, it enhances clathrin release by increasing the ATPase activity of HSC70, enhancing the efficiency of the clathrin-mediated endocytosis pathway to promote BRSV entry.

We initially demonstrated that HSPA4 could promoted BRSV replication by overexpressing and silencing HSPA4 in MDBK cells, followed by inoculation with BRSV. A recent report suggested that HSPA5, a member of the Hsp70 family, may play a role in the replication, attachment, and entry of PEDV [37]. In light of these findings, we also investigated the involvement of HSPA4 in BRSV entry and attachment. Our findings indicate that HSPA4 specifically facilitates the entry of BRSV into cells but does not participate in the attachment process. This distinction may be attributed to the cellular localization of HSPA4, which is primarily restricted to the cytoplasm and nucleus [36], whereas HSPA5 is more ubiquitously distributed in the cytoplasm, nucleus, and cell membrane [38,39]. During the early stages of PEDV infection, the interaction between PEDV S protein and HSPA5 on the cell membrane promoted PEDV attachment [37], but HSPA4 does not have the ability to interact with virus proteins.

Endocytosis is a common route through which viruses enter host cells [7]. Previous research has indicated that BRSV enters into MDBK cells through clathrin-mediated endocytosis [12]. In this study, we investigated the impact of clathrin-mediated endocytosis on HSPA4-facilitated BRSV entry by utilizing chlorpromazine, an inhibitor of this pathway. Our findings revealed that HSPA4 enhances BRSV entry by modulating clathrin-mediated endocytosis. Specifically, CHC, a crucial functional domain of clathrin, was found to be regulated by HSPA4, suggesting a potential role of HSPA4 in promoting CHC expression. Additionally, during the early stages of BRSV infection in MDBK cells, the activation of the PI3K–Akt and Src–JNK signaling pathways was observed to boost CHC expression, thereby strengthening clathrin-mediated endocytosis [12]. HSPA4 has been associated with various cancers, including glioma, and a KEGG enrichment analysis revealed that HSPA4 is linked to the PI3K–Akt signaling pathway [22]. Consequently, we explored the influence of HSPA4 on the PI3K–Akt pathway and found that HSPA4 could modulate the expression of p-PI3K and p-Akt. We subsequently investigated the role of the PI3K–Akt pathway in the HSPA4-mediated regulation of BRSV entry using wortmannin and Akti-1/2, which are inhibitors of PI3K and Akt. Our results conclusively demonstrated that HSPA4 facilitates BRSV entry into MDBK cells by regulating the PI3K–Akt signaling pathway.

HSC70, a molecular chaperone protein, plays a crucial role in the regulation of clathrin-mediated endocytosis. Acting as a clathrin-uncoating ATPase, it interacts with clathrin to modulate clathrin-coated assembly and disassembly, ultimately enhancing clathrin-mediated endocytosis [23,24]. Additionally, HSP110 accelerates the ATPase cycle of HSC70 by binding to the ATPase region of HSC70, facilitating ADP release and ATP binding, thereby promoting clathrin-mediated endocytosis [25,26]. It is hypothesized that HSPA4 may also enhance clathrin-mediated endocytosis by promoting the ATPase cycle of HSC70. Our experimental results demonstrated that HSPA4 can interact with HSC70 and CHC interacts with HSC70 in MDBK cells. We used apoptozole and VER155008, which are inhibitors of HSC70 ATPase activity [31,32], to investigated the role of HSC70 in HSPA4 promoting BRSV entry. The findings revealed that HSPA4 facilitated the clathrin-mediated endocytosis of BRSV into MDBK cells by modulating the ATPase activity of HSC70.

This study investigated the role of HSPA4 in facilitating the entry of various viruses into host cells. The findings demonstrated that HSPA4 not only upregulated CHC expression in Vero and PK15 cells, but it also facilitated the entry of BoHV-1, BPIV3, PEDV, and TGEV into host cells. These results suggest that HSPA4 can enhance clathrin-mediated endocytosis in different cells, thereby promoting the entry of a variety of viruses. The research indicates that HSPA4 could potentially serve as a target for developing resistance against viral infections.

**Figure 8:**
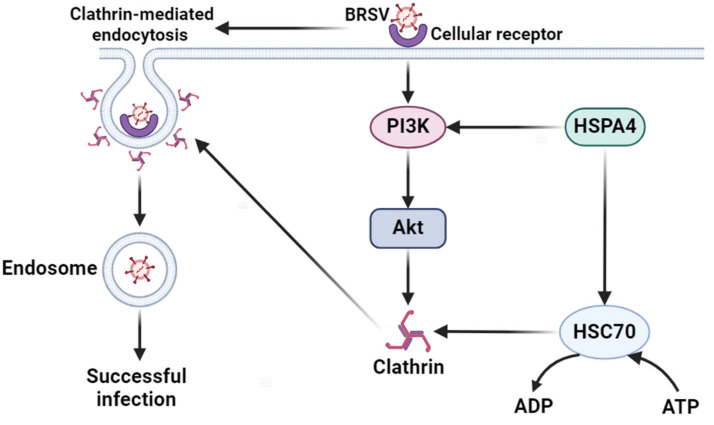
Model of mechanism through which HSPA4 promotes BRSV entry. The activation of the PI3K–Akt signaling pathway by HSPA4 upregulates CHC expression, thereby boosting clathrin-mediated endocytosis and promoting BRSV entry. Additionally, HSPA4 strengthens HSC70 ATPase activity to enhance the combination of ATP and HSC70, leading to the release of clathrin and improving the efficiency of clathrin-mediated endocytosis, further enhancing BRSV entry.

In conclusion, BRSV can enter MDBK cells via clathrin-mediated endocytosis during the early stages of infection. Concurrently, the interaction between BRSV and cell receptors activates the PI3K–Akt signaling pathway, which promotes clathrin expression and clathrin-mediated endocytosis. HSC70 functions as a clathrin-uncoating ATPase and interacts with clathrin to regulate both the assembly and disassembly of clathrin-coated vesicles, thereby enhancing clathrin-mediated endocytosis. In this process, HSPA4 can facilitate the activation of the PI3K–Akt pathway, increase HSC70 ATPase activity, and further promote the entry of BRSV into cells (Figure 8).

## Figures and Tables

**Table 1:** The sequences of the siRNAs, primers, and probe.

Name	Sequence (5′–3′)
HSPA4-siRNA1	GGTTCCTTGTTTCTATACT
HSPA4-siRNA2	CAGCTGAAGAAGGGTCAAG
HSPA4-siRNA3	ACTCTTGAGGCTTATTATA
BRSV-N-qF	TGAAAAGYACCCTCATTACAT
BRSV-N-qR	CATCACTTGACCTGCTCCAT
BRSV-N-probe	TGCAGGGTTATTCATGAATGCATATGGA

## Data Availability

The original contributions presented in the study are included in the article. Further inquiries can be directed to the corresponding authors.
